# Mineral Indicators of Geologically Recent Past Habitability on Mars

**DOI:** 10.3390/life13122349

**Published:** 2023-12-15

**Authors:** Roger Hart, Dawn Cardace

**Affiliations:** 1 Department of Physics and Engineering, Community College of Rhode Island, Lincoln, RI 02865, USA; 2 Department of Geosciences, University of Rhode Island, Kingston, RI 02881, USA; cardace@uri.edu

**Keywords:** serpentinization, Mars, astrobiology

## Abstract

We provide new support for habitable microenvironments in the near-subsurface of Mars, hosted in Fe- and Mg-rich rock units, and present a list of minerals that can serve as indicators of specific water–rock reactions in recent geologic paleohabitats for follow-on study. We modeled, using a thermodynamic basis without selective phase suppression, the reactions of published Martian meteorites and Jezero Crater igneous rock compositions and reasonable planetary waters (saline, alkaline waters) using Geochemist’s Workbench Ver. 12.0. Solid-phase inputs were meteorite compositions for ALH 77005, Nakhla, and Chassigny, and two rock units from the Mars 2020 Perseverance rover sites, Máaz and Séítah. Six plausible Martian groundwater types [NaClO_4_, Mg(ClO_4_)_2_, Ca(ClO_4_)_2_, Mg-Na_2_(ClO_4_)_2_, Ca-Na_2_(ClO_4_)_2_, Mg-Ca(ClO_4_)_2_] and a unique Mars soil-water analog solution (dilute saline solution) named “Rosy Red”, related to the Phoenix Lander mission, were the aqueous-phase inputs. Geophysical conditions were tuned to near-subsurface Mars (100 °C or 373.15 K, associated with residual heat from a magmatic system, impact event, or a concentration of radionuclides, and 101.3 kPa, similar to <10 m depth). Mineral products were dominated by phyllosilicates such as serpentine-group minerals in most reaction paths, but differed in some important indicator minerals. Modeled products varied in physicochemical properties (pH, Eh, conductivity), major ion activities, and related gas fugacities, with different ecological implications. The microbial habitability of pore spaces in subsurface groundwater percolation systems was interrogated at equilibrium in a thermodynamic framework, based on Gibbs Free Energy Minimization. Models run with the Chassigny meteorite produced the overall highest H_2_ fugacity. Models reliant on the Rosy Red soil-water analog produced the highest sustained CH_4_ fugacity (maximum values observed for reactant ALH 77005). In general, Chassigny meteorite protoliths produced the best yield regarding Gibbs Free Energy, from an astrobiological perspective. Occurrences of serpentine and saponite across models are key: these minerals have been observed using CRISM spectral data, and their formation via serpentinization would be consistent with geologically recent-past H_2_ and CH_4_ production and sustained energy sources for microbial life. We list index minerals to be used as diagnostic for paleo water–rock models that could have supported geologically recent-past microbial activity, and suggest their application as criteria for future astrobiology study-site selections.

## 1. Introduction

Serpentinization and water–rock reactions on rocky ocean worlds with subsurface silicates interacting with subsurface water layers are increasingly of interest as drivers of planetary habitability [1,2,3,4,5,6,7]. Serpentine group minerals have also been detected in the study of asteroids [8], comets [9], and meteorites, especially in some carbonaceous chondrites [10]. Serpentinization relies on available parent minerals in the olivine and pyroxene groups being exposed to water and oxidative alteration through time, producing serpentine minerals and associated phases. 

Ultramafic parent rocks produce serpentine-rich alteration assemblages; mafic parent rocks produce albite- and phyllosilicate-rich alteration assemblages, with minor serpentinization. In both cases, rocky solids transform, causing geochemical changes in co-existing aqueous solutions, and may release H_2(g)_ and diverse hydrocarbon-containing compounds over time. Biologically useful hydrogen is produced as Fe^2+^ in olivine is oxidized and H atoms in H_2_O are reduced, yielding Fe oxides and free H_2_ [10]. H_2_ production via low-temperature (30 °C to 70 °C) water–rock reactions has been examined through laboratory studies [11], but are being examined more comprehensively [12]. 

CH_4_ has been identified in both mafic and ultramafic rock units on Earth, predominantly in ophiolites and other settings across various spatiotemporal settings (Supplementary Table S1, references therein, based on [13]). CH_4_ and more complex hydrocarbons can be produced from CO_2_ and H_2_ and through Fischer–Tropsch Type (FTT) synthesis [14,15,16,17]. However, FTT reactions cannot be the sole drivers of organic synthesis in planetary materials, given other processes illustrated in the Stecker synthesis, spark discharge experiments, and ultraviolet irradiation studies [18,19]. 

Examples of reactions of serpentinization resulting in astrobiologically relevant species include reactions presented by Oze and Sharma [20], which include reactions (1)–(3):

6Fe_2_SiO_4_ + 7H_2_O = 3Fe_3_Si_2_O_5_(OH)_4_ + Fe_3_O_4_ + H_2(aq)_ (1)

12FeSiO_3_ + 2H_2_O + CO_2 (aq)_ = 4Fe_3_O_4_ + CH_4_ + 12SiO_2(aq)_ (2)

12FeSiO_3_ + 2H_2_O + CO_2 (aq)_ = 4Fe_3_O_4_ + CH_4_ + 12SiO_2[Amorphous Silica]_ (3)

Key gas-phase reactions associated with these processes are shown in reactions (4) and (5):

4H_2_ + CO_2_ = CH_4_ + 2H_2_O (4)

H_2_ + SO_2_ = H_2_S + O_2_ (5)

Subsurface mafic–ultramafic rock alteration processes arguably produce H_2_ and CH_4_ over long time periods, and provide sheltered niches for life, like porous voids, and the preservation of biosignatures. At the same time, mafic and ultramafic rocks on Mars are well- constrained from the meteorite record [21,22,23,24,25,26] and recent rover reports from Jezero Crater sites [27,28,29]. Further, plausible paleo groundwater chemistries for Mars have been rigorously vetted [30,31,32,33,34,35,36,37,38,39,40,41,42,43]. Diverse alteration products of water–rock reactions have been documented on Mars [26,44,45,46,47,48,49,50,51,52,53]. Alteration minerals record environmental change and geochemical transitions, serving as proxy data for changing (geologically controlled) habitability [51,54,55,56,57]. 

Increasingly well-resolved surface mineralogy data for Mars (buttressed by rover-derived data streams) provide an excellent basis for models of water–rock interactions. For example, olivine- and pyroxene-bearing material has been mapped in the Nili Fossae region [58,59]. In this ~30,000 km^2^ region on Mars, a large depression has been described as mélange terrain (elevation −0.6 km, Syrtis Major quadrangle, ~22° N, ~75° E) [46]. Olivine in the Nili Fossae protolith ranges compositionally from 30 to 70% fayalite (Fe_2_SiO_4_, [47]); prior work identified surface olivine and a generally basaltic composition [48]. Higher percentages of fayalite produce stoichiometrically more molecular hydrogen via serpentinization-driven reactions, for example reaction (1). Complementary investigations targeting Nili Fossae olivine using the OMEGA spectrometer on Mars Express [60,61] identified adjacent phyllosilicate deposits [44,62], and serpentine has been confirmed [46,50,63,64,65]. Spatially related smectite, carbonate and serpentine found in this area likely correspond to discrete alteration events [66]. 

Presently, the Mars 2020 Perseverance rover is collecting data near its Jezero Crater landing site, seeking signs of past life [67,68,69,70]. Prior scientific work assessed the astrobiological potential of ancient Mars, surface geological properties and processes, and Martian habitability [71,72,73,74,75,76]. Recent discoveries by the Perseverance rover during the Mars 2020 mission have characterized two basement igneous rock units, one mafic unit (Máaz) and a deeper ultramafic unit (Séítah), which show evidence of aqueous alteration [27]; spectral data for the Séítah unit suggest talc or serpentine [77] This Noachian-aged basin [78] likely experienced aqueous alteration between 3.8 and 3.2 Ga [78,79,80], possibly as a lake-like system [80,81,82]. We aim to provide a theoretical model of possible mineralogy, associated with plausible habitability for a better targeting of further exploration of the Martain surface and near-subsurface.

Mindful of sustained interest in assessing the habitability of the Martian near-subsurface and future mineralogical datasets for material sampled by Perseverance, we model the reactions of three Martian meteorites and the Máaz and Séítah lithologies with plausible Martian waters. In this work, we examine the geochemical evolution of plausible subsurface pore- and/or fracture-network environments, which conceptually correspond to Nili Fossae rock units located proximal to the Jezero Crater. Modeling inputs (rock and water types) are well supported by existing data, and modeling outputs (secondary minerals and altered waters/evolved gases) are thoughtfully compared to modern observations. Surveying the products of water–rock alteration models, we isolate mineral tracers of specific geologically recent paleo water–rock interactions. 

Further, we assess the spontaneity of four common microbial metabolic reactions in Mars-relevant mafic–ultramafic systems, to determine which mineral indicators correspond to specific paleo habitats on Mars. Diagnostic secondary minerals for specific water–rock reaction paths ranged from singular phases to unique sets of minerals. Most simply, a single diagnostic phase is keyed to a reaction path model; the mineral saponite-Na is related uniquely to the Séítah reactions in the Rosy Red simulated fluid. A specific set of minerals defines the Séítah reactions with the NaClO_4_ type of water: key products are natrolite with phlogopite. In other cases, more minerals constitute the diagnostic sets that correspond to specific water–rock reaction paths. In all, we provide new model results that underscore the importance of the co-occurrence of serpentine and saponite as indicator minerals in remotely sensed or rover-based data streams, as signaling promising sites for astrobiological investigation.

## 2. Materials and Methods

We modeled aqueous alteration of mafic and ultramafic rocks using The Geochemist’s Workbench (www.gwb.com (1 December 2023)), 12th Edition, without selective phase suppression, to determine solid–liquid–gas–phase equilibrium species [83] in the near-subsurface on Mars.

### 2.1. Water–Rock Geochemical Modeling

#### 2.1.1. Code and Database

Solute activity was calculated using the extended Debye–Hückel B-dot equation, useful for ionic strengths of up to 3 molal solutions, from 273 K to 573 K [83,84,85]. We used the Geochemist’s Workbench modeling platform, to extend prior water–rock modeling in planetary contexts [3,4,5,6,7,66,86,87,88,89,90,91,92,93,94]. The extended Debye–Hückel B-dot equation was engaged, since all modeled ionic strengths considered here are between 0.0407 and 3.435 molal at *ξ* = 1. Eh ranges for all simulations are between −893.0 and −208.0 mV, is reducing, and generally similar to, some terrestrial groundwaters, high-salinity waters, or water–rock systems isolated from our oxygen-rich atmosphere.

We utilized the thermodynamic database from thermo.com.V8.R6+.tdat, distributed at https://www.gwb.com/data/thermo.com.V8.R6+.tdat (1 December 2023) [95]. Temperatures were held constant, modeled at 373.15 K, simulating recent-geologic-past magmatic-, impactor-, or radionuclide-derived heat sources (e.g., small-to-medium craters ~20–30 km in diameter produce a range of ~373 to 393 K [96] or radionuclides [2,4]). Pressure was constant at 1.013 kPa. Generally, water/rock ratios were ~1:1 kg, with the addition of solutes increasing the mass of the water slightly; this is a relatively low water–rock ratio, hypothesized to have occurred in subsurface or lacustrine environments in the geologically recent past [41,97,98,99,100,101,102]. 

Limitations exist in every modeling simulation. We recognize that model inputs are bulk rock representations, lacking fine-scale mineralogy or mineral chemistry; as such, the models are more broadly applicable to mafic–ultramafic-hosted systems. We assume that the reactant water aptly represents the recent-geological-past natural waters on Mars/in Martian meteorites, leveraging the critical works of Toner et al. [103,104,105] and Leong and Shock [13], to validate this choice. We applied an equilibrium modeling approach, reacting all available reagents, in an effort to consider the changing distribution of phases as equilibrium is approached; in nature, a flow-through system could replenish limiting reagents (e.g., through diffusion or advection). Also, we would state that the thermodynamic database used in this work did not account for all mineral solid solutions across all compositions [7,83].

Modeling experiments are always fundamentally constrained by the boundaries of the selected thermodynamic database, which establishes the mineral phases to be considered in any derivative model. We did not deliberately suppress any phases, and share all model outputs. We carefully interpret reaction-path-modeling outputs by evaluating minerals not likely to form in terrestrial-planet geological conditions, based on conventional geological principles, and consider the broader impacts of the chemical inventory of the system, to safeguard the applicability of the results. 

#### 2.1.2. Geophysical Considerations

The modeled environment is appropriate for depths greater than ~5 m below the surface of Mars, at low latitudes. The Nili Fossae experience a Martian equatorial climate [106], and temperatures measured by the Thermal Emission Imaging System (THEMIS) are between ~250 and 300 K [59]. This is in the range of the freezing point of a 3–5% saltwater solution with density of ~1035 kg/m^3^ [36,38,39,43,84,103]. In addition, the subsurface mafic–ultramafic rock unit at the Nili Fossae (taken at ~9 m’ depth) has enough radiation shielding to allow life; at this depth, cells and biological debris would be beyond the incident electromagnetic and energized-particle irradiated zone, which would ionize biological molecules and render the environment sterilized [107,108]. The current depth of this sterilization is estimated at 2 to 4 m’ depth beneath the Martian regolith, depending on the density of the overlying material; the maximum depth depends on assumptions regarding near-surface Martian soil properties and a deeper mafic–ultramafic unit [108]. We model water-rock reactions at temperatures and pressures relevant to these clement subsurface horizons, as putative habitats.

#### 2.1.3. Input Conditions in Modeling: Solids

Observed rock-forming silicates on Mars and associated weathering products are consistent with model inputs. Primary olivines have been detected on Mars [28,58,60,109,110,111,112,113,114,115,116,117], as have clinopyroxenes [60,116]. 

The olivine-rich surface layer observed at the Nili Fossae on Mars overlays a phyllosilicate basement [60,77,109,110,111,118]; basement rocks were likely severely fractured by ancient impacts, with possible extensive alteration. This region is proximal to the altered, basaltic rocks of the Máaz formation, which exhibits FeO values of ~20 wt %, and the high-olivine ultramafic rock unit, with FeO values of ~30 wt % [28].

Perseverance rover-based data for two sites on the Martian surface are used to structure two specific reaction paths. Mineralogical evaluation of the Máaz site by Farley et al. [27] suggests the presence of augite, plagioclase, ilmenite, ferrosilite, and unidentified iron oxide solids. Materials observed at the Séítah site on Mars show the presence of olivine, augite with minor feldspars, phosphates, and Cr- and Ti-bearing Fe oxide solids [27].

Regarding the meteorite record, SNC meteorites are classified as mafic-to-ultramafic rocks of Amazonian age, between ~3.0 Ga, and the present [119,120,121,122,123,124,125,126]. Major SNC meteorite minerals resemble terrestrial mafic igneous rock compositions (olivine, plagioclase, clinopyroxene, and orthopyroxene [126]). Minor minerals include shock glasses, Cr-spinel, sulfide minerals, phosphates, titanomagnetite, ilmenite, zircon and silica minerals [126]. 

Martian meteorites (shergottites, nakhlites, and chassignites, together known as ‘SNC meteorites’), are rare samples of Mars’s minerals, geochemical inventories, and petrological textures. SNC meteorites are essentially samples of the Martian crust or mantle ([126]; references therein), and are investigated with respect to volcanic processes, the alteration, structural and stratigraphic analysis of igneous processes, the volatile content of the subsurface, and impact event processes. With the return of samples from the Mars 2020 Perseverance mission, unparalleled mineralogical and geochemical analyses are possible for target Martian rocks with specific locality information [74]. These meteorite groups have also had extensive associated geochemical modeling (e.g., [6,86,87,89,127,128,129,130]) to which this work can be compared.

**Figure 1:**
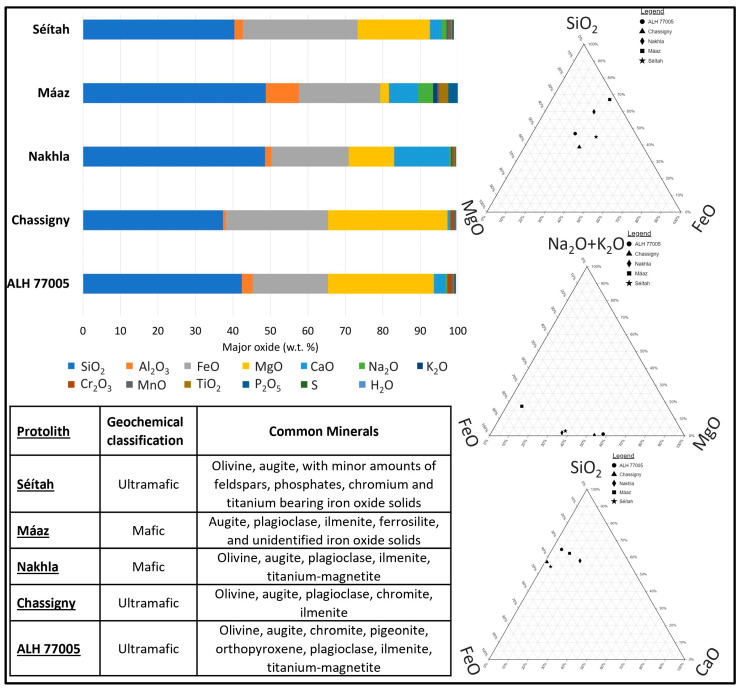
Initial protolith inputs for reaction path modeling showing their major oxide w.t. % of ALH77005 (lherzolitic shergottite, Chassigny (Chassignite type), Nakhla (Nakhlite type)), Perseverance sites, Máaz and Séíta, and three common ternary plots, SiO_2_-MgO-FeO on top, Na_2_O+K_2_O-FeO-MgO in the middle, and SiO_2_-FeO-CaO on the bottom. Chassigny, Séítah, and ALH77005 all exhibit between 48 and 59 w.t. % FeO+MgO values, and are considered ultramafic. Nakhla and Máaz are considered mafic. Nakhla shows an approximate FeO+MgO value of 33%, and Máaz a value of ~24%. (Supplementary Figure S1 displays how PC1 is largely controlled by MgO (approx. −0.85, SiO_2_ (approx. 0.34), and CaO (approx. 0.31), and PC2 is largely controlled by FeO (approx. −0.65), CaO (approx. 0.51), and MgO (approx. 0.36)). Note that the subset table shows protolith geochemical classifications and major minerals, as reported for the meteorites [21] and the two Perseverance sites [27].

Here, data for the Máaz and Séítah research sites, as well as for selected meteorite types, were input as average whole-rock compositions (Figure 1). Input data are provided in Figure 1 and Supplementary Table S2 for ALH77005 (lherzolitic shergottite), Chassigny (chassignite type), and Nakhla (nakhlite type), all from Lodders [21] and the Perseverance rover research sites, Máaz and Séítah (as reported in [27]). 

#### 2.1.4. Input Conditions in Modeling: Fluids

Martian groundwater inputs derive from Toner et al. [103], informed by other significant works [37,42,131,132], incorporating (i) the detection of chlorate during the Mars polar Phoenix mission [36,133,134] and (ii) possible evidence of subglacial water at the Martian pole via Mars Express spacecraft radar evidence [135]. Martian groundwater studies [40,42,136] and the premise that modern Mars may exhibit a perchlorate-type water that has high levels of salinity [43,103,137,138] influenced water chemistry inputs in this work. Taken together, even in the harsh, modern Martian environment, it is possible that subsurface waters could support biological processes [137,138,139,140]. Microscopic-scale subzero water in chemical reactions like peroxide decomposition and sulfate formation, not considered here, is important for astrobiology [30]. 

Initial water compositions were generally characterized by the following chemistry: NaClO_4_, Mg(ClO_4_)_2_, Ca(ClO_4_)_2_, Mg-Na_2_(ClO_4_)_2_, Ca-Na_2_(ClO_4_)_2_, and Mg-Ca(ClO_4_)_2_ [105]; these waters have perchlorate as the dominant anion and have freezing-point depression properties [30,104,137]. Salts expected to occur in regolith materials have been identified on Mars (cf. [33,38,141]. In parallel, a soil-water analog solution based on digestion of Mars regolith from Rosy Red, a Phoenix Lander site [103], served as another input (“Rosy Red”). All were considered under present-day Martian atmospheric properties [142] (comprehensive list of properties in Supplementary Table S3). 

### 2.2. Statistical Analysis of Modeled Results

Statistical discrimination of relationships in model outputs focused on equilibrium mineral assemblages, since minerals outlast aqueous or gas phases. We utilized the statistical analysis program JMP^®^, Version Pro 16. SAS Institute Inc., Cary, NC, USA, 1989–2023, applying robust multivariate principal component analysis (RPCA) to cleaned data [136,143,144,145]. Statistical treatment was based on data covariances [145,146,147]. Eigen decomposition was applied to the covariance matrix to determine the eigenvalues of the modeled dataset, and allow its interpretation [146,147]. 

### 2.3. Bioenergetic Computations

Bioenergetic modeling (as in [148,149,150,151]) quantifies Gibbs Free Energy (Δ*G_r_*) values for reactions of interest. Δ*G_r_* is the sum of the standard-state Gibbs Free Energy value (Δ$G_{r}^{o}$) at the conditions of interest (373.15 K, associated with residual heat from an impact event or magmatic system, and 101.3 kPa, similar to <10 m depth), and the product of the gas constant (R), temperature (T, in Kelvin), and the natural log of the activity product ($Q_{r}$), as in Equation (6):

(6) $$\left(\frac{\Delta Gr}{d\xi }\right)=∆G_{r}^{o}+RT\;lnQ_{r}\;.$$

The activity product, $Q_{r}$, was computed using the activity data for ith species, $a_{i}$, and $v_{i,r}$, the stoichiometric reaction coefficient of the ith species, as in Expression (7):

(7) $$Q_{,r}=\prod a_{i}^{v_{i,r}}\;$$

When Δ*G_r_* is negative, by convention, the modeled reaction is thermodynamically favorable; we infer a feasible microbial metabolic reaction, or a habitable niche. When the system is at constant temperature and pressure, Δ*G_r_* varies with the extent of the chemical reaction (*ξ*), as the activities of relevant species shift. Activity data from GWB output files were used in calculations (Supplementary Table S3).

Four generalized microbial metabolic reactions were targeted in this study, as in previous work [150,151,152,153,154,155,156]. The Gibbs Free Energy computation values for each reaction (kJ/mol) were normalized to the number of electrons (e^−^) transferred [156]. These reactions are known to support microbial activity in Earth environments, and represent key carbon transformations that may be important for Martian settings:

sulfate reduction [SO_4_^2−^+ 4H_2(aq)_ +2H^+^ = H_2_S_(aq)_ + 4H_2_O_(l)_]; (8)

methanotrophy [CH_4(aq)_ + SO_4_^2−^ + H^+^ = HCO_3_^−^ + H_2_S_(aq)_ + H_2_O_(l)_]; (9)

methanogenesis [CO_2(aq)_ + 4H_2(aq)_ = CH_4(aq)_ + 2H_2_O_(l)_]; and (10)

anaerobic methane oxidation, or AMO [SO_4_^2−^ + 2H^+^ + CH_4(aq)_ = CO_2(aq)_ + H_2_S_(aq)_ + 2H_2_O_(l)_] (11)

## 3. Results

### 3.1. Bulk-System Geochemistry

We modeled the reactions of the five protoliths with seven plausible planetary waters, resulting in different equilibrium solid, aqueous, and gas assemblages. Inputs are provided in Supplementary File S1. Our results show that (i) modeled solid products reproduce many minerals known to be part of Martian (near-)surface materials, and (ii) some modeled product waters have higher total dissolved solids than are typically observed in terrestrial serpentinites, while pH values are within the range of continental serpentinization systems, such as in Oman, Coast Range, Del Puerto, and Bay of Islands Ophiolites (Supplementary Table S1). 

**Figure 2:**
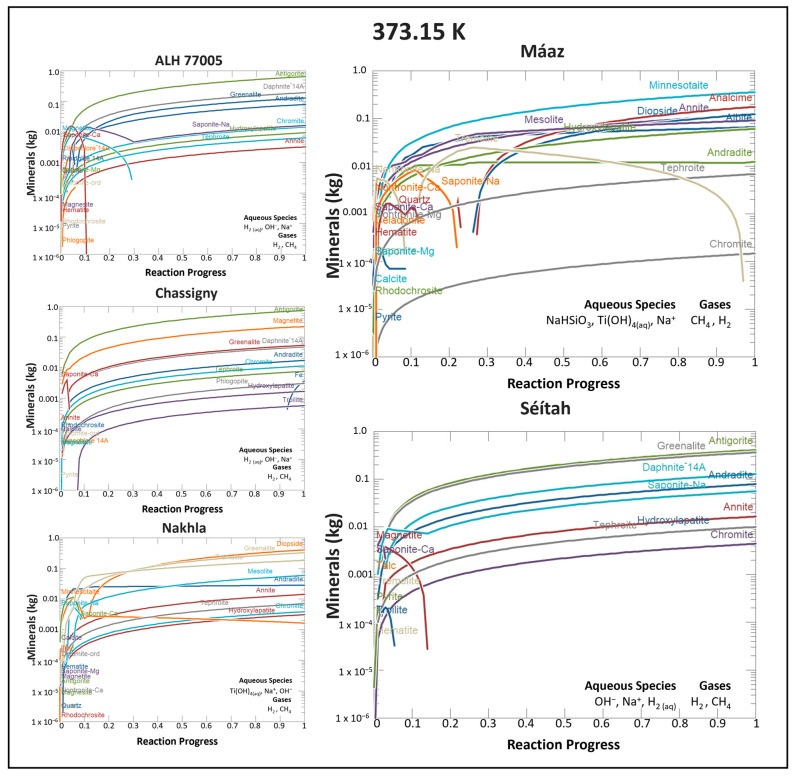
Representative charts of reactions with the modified Rosy Red Mars Phoenix Lander simulant water. Meteorite protoliths (ALH77005, Chassigny, and Nakhla) and Mars 2020 Perseverance rover sites (Máaz and Séítah) reacted at 373.15 K. All plots show the major minerals produced (kg) on the vertical axis; inset text shows dominant species at equilibrium in aqueous and gas phases. Equilibrium minerals, waters, and gases differ. Specifically, Máaz yields a minnesotaite–mesolite-annite assemblage (pyrophyllite- and mica-bearing, with zeolites) and Séítah models yield an antigorite–greenalite–daphnite–andradite assemblage (dominated by serpentine, chlorite, and hydrogarnet groups). Further, the expected aqueous Cl^−^ and H_2(aq)_ values diverge, and H_2_:CH_4_ ratios vary between models (see supplementary documents for specific values).

Water and protolith chemistries exert important controls on serpentinization-related reaction products (Figure 2). For example, in all 373 K models, modeled reaction paths drive pH variation. Starting solutions (*ξ* = 0) were all acidic, ranging from pH ~ 5.04 to 5.286; equilibrium solutions (*ξ* = 1) were mostly basic, except for two simulations that retained acidic pH values. Recall that the pH of neutrality at 373 K is 6.13, not 7. The Máaz reaction path with Mg(ClO_4_)_2_ water yielded a final solution pH of 5.27 (acidic), and the Máaz reaction path with CaMg(ClO_4_)_2_ water yielded a final solution pH of 5.39 (acidic). For comparison, the Chassigny reaction path with CaMg(ClO_4_)_2_ water resulted in a final solution pH of 6.4 (basic), and Chassigny reacted with Na_2_Mg(ClO_4_)_2_ water resulted in a final solution pH of 6.6 (basic); Nakhla reacted with Mg(ClO_4_)_2_ water resulted in a final solution pH of 6.7 (basic). The more corrosive (acidic) solutions were generated by the Máaz-based models.

The dominant product mineral by mass (>100 g formed) in many models was the mineral antigorite (a serpentine, general composition Mg_3_Si_2_O_5_(OH)_4_ [147]). Responding to temperature, system chemistry, and oxygen fugacity (*f*O_2_), other important product minerals included iron-rich oxides (hematite, magnetite) and other phyllosilicates (greenalite (a mixed-valence state Fe-serpentine), cronstedtite (a mixed-valence state Fe-serpentine, with some Fe substitution for Si), saponite (calcium-rich trioctahedral smectite), daphnite (magnesian chamosite, a chlorite mineral), and andannite (Fe-rich biotite)). Hydroxylapatite (OH-apatite, a phosphate mineral, and andradite (garnet group, often hydrated in model outputs) were repeatedly observed. 

Several solid phases were predicted by the thermodynamic modeling, but are not likely to be stably produced at 373 K (100 °C) in quasi-diagenetic alteration conditions. These phases are either pure metallic solids such as “Fe_(s)_” or rock-forming primary minerals, including “FeO” (an ideal ferrous iron oxide, not necessarily the mineral wüstite), “tremolite” (amphibole group, a Ca- Mg-silicate), “diopside” and “hedenbergite” (clinopyroxene endmember minerals, Ca- Mg- Fe-silicates with variable cation substitution), “tephroite” (Mn-silicate), and “chromite” (an Fe- Cr-oxide mineral, in the spinel group). These modeled product phases should be considered as chemical placeholders, showing that an equilibrium-phase assemblage, which includes, for example, a diopside, would necessarily (stoichiometrically) use up the available Ca, Mg, Fe, Mn, Cr, Si, and O in the system. Diverse phyllosilicates and metastable mineral intermediaries are possible, though not in the selected thermodynamic database, and thus not queried by our model. Tremolite, for example, has not been empirically observed as a secondary phase. 

Common terrestrial secondary minerals in mafic rock weathering (largely phyllosilicates) and zeolite or seafloor diagenetic minerals are eminently reasonable model outputs. Other solid phases that repeat in model outputs, but that often occur in higher temperature/magmatic systems found on Earth, include (hydroxyl)apatite, hydrogarnets, troilite, annite, and quartz; these phases, though less common, are supported in the diagenetic literature, and are regarded also as valid model results.

Important aqueous products include Cl^−^, Na^+^, Ca^2+^, H_2(aq)_, Mg^2+^, CH_4(aq)_, HCO_3_^−^ and CO_3_^2−^_(aq),_ SO_4_^2−^_(aq)_. Dominant gases produced in these simulations were H_2(g)_ and CH_4(g)_, with minor H_2_O_(g)_.

#### 3.1.1. Key Mineral Products: Martian Meteorite-Based Models

Modeling based on the three meteorite reactants yielded differing results. The phyllosilicate minerals greenalite and daphnite were produced in abundances greater than 0.25 moles (at *ξ* = 1) for models with ALH 77005, Nakhla, and Chassigny. Minor minerals produced in each reaction path differed from model to model (see Supplementary File S1). 

Reaction paths with ALH77005 commonly produced antigorite (~0.159 to 0.172 moles), daphnite-14Å (~0.298 to ~0.309 moles), and greenalite (~0.358 to ~0.445 moles). To lesser extents, “chromite” (~0.068 to ~0.071 moles) and hydroxylapatite (~0.064 to ~0.071 moles) were also produced in all models. Andradite garnet (~0.057 to ~0.178 moles) and “tephroite” (~0.033 to ~0.0035 moles) were produced in all reaction paths except with Mg(ClO_4_)_2_-type water. Troilite was produced in all reaction paths except with NaClO_4_-type water. Annite was only produced in the NaClO_4_ and Rosy Red models. “Diopside” (~0.068) was produced in only the Ca(ClO_4_)_2_ and CaNa_2_(ClO_4_)_2_ models. Magnetite (~0.006 to ~0.02) was produced in the CaMg(ClO_4_)_2_, Mg(ClO_4_)_2_ and Na_2_Mg(ClO_4_)_2_ models. Saponite was only produced in the NaClO_4_ model. 

All reaction paths with Chassigny yielded the silicate minerals antigorite (~0.164 to ~0.183 moles), daphnite-14Å (~0.066 to ~0.077 moles), hydroxylapatite (~0.0033 to ~0.0037 moles), and greenalite (~0.142 to ~0.191 moles). Oxide minerals such as “chromite” (~0.048 to ~0.056 moles) and magnetite (~0.83 and ~1.01 moles) were produced, along with additional “elemental Fe” (~0.29 to ~0.43 moles). Andradite garnet (~0.033 to ~0.045 moles) was produced in the Ca(ClO_4_)_2_, CaNa_2_(ClO_4_)_2_, NaClO_4_, and Rosy Red models, and phlogopite (~0.0072 to ~0.0086 moles) was produced in NaClO_4_ and Rosy Red models. Troilite (~0.0064 to ~0.089 moles) was produced in all cases. “Tephroite” (~0.037 to ~0.041 moles) was produced in Ca(ClO_4_)_2_, CaNa_2_(ClO_4_)_2_, NaClO_4_, and Rosy Red models. 

In the Nakhla reaction paths, “diopside” was the dominant mineral produced, in terms of mass. Other minerals included andradite (~0.022 to ~0.035 moles), annite (~0.027 to ~0.031 moles), hydroxylapatite (~0.0060 to ~0.0067 moles), greenalite (~0.886 to ~1.009 moles), and chromite (~0.017 to ~0.019 moles). “Tremolite” was produced in all reactions (~0.198 to ~0.684 moles) except Rosy Red. “Diopside” (~1.48 to ~2.30 moles) and mesolite (~0.151 to ~0.167 moles) were produced in all models except the Mg(ClO_4_)_2_ model. The Ca(ClO_4_)_2_ and Rosy Red reactions produced minnesotaite (~0.0005 to ~0.005 moles). Antigorite (~0.039 moles) and magnetite (~0.032 moles) were only produced in the Mg(ClO_4_)_2_ reaction. Prehnite was only produced in the Ca(ClO_4_)_2_ reaction (~0.014 moles). Saponite-Ca was only produced in the Mg(ClO_4_)_2_ reaction (~0.199 moles) and Saponite-Na was only produced in the NaClO_4_ reaction (~0.015 moles).

#### 3.1.2. Key Mineral Products: Jezero Crater Protolith Models

**Figure 3:**
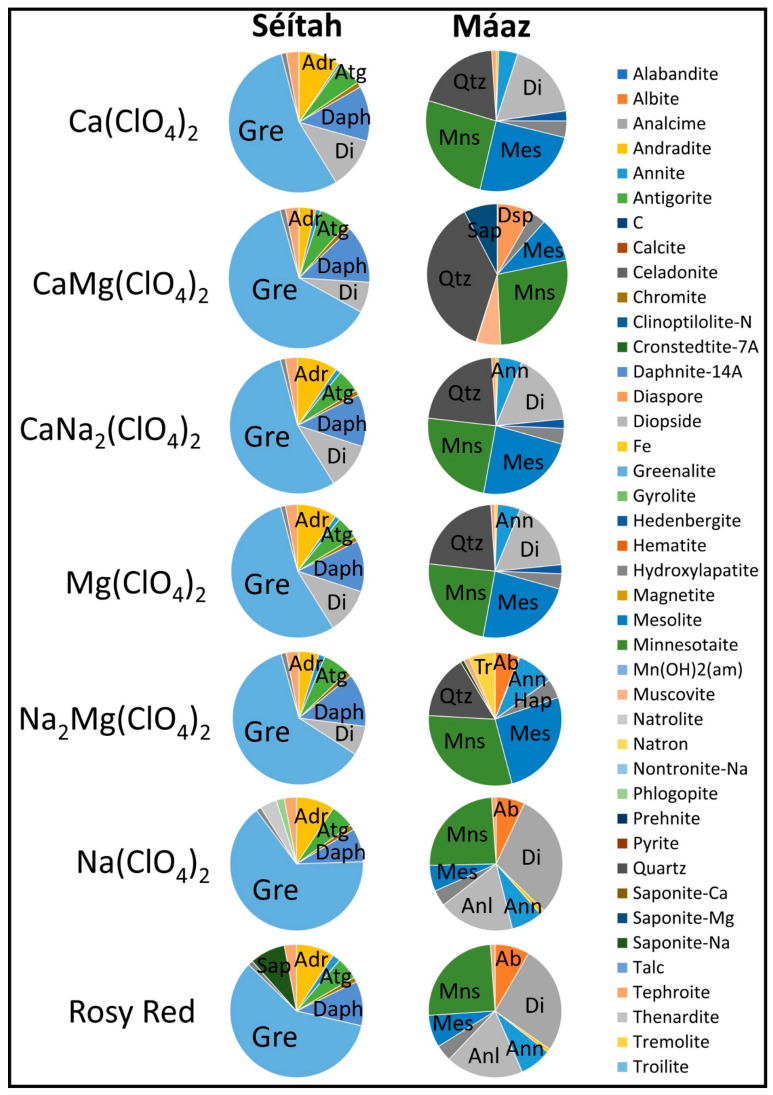
Predicted mineral assemblages at equilibrium as pie charts of molar abundances for the Séítah and Máaz models, with a suite of reasonable planetary waters, at 373.15 K. Major mineral phases are labelled using traditional conventions: Albite (Ab), Analcime (Anl), Annite (Ann), Andradite (Adr), Antigorite (Atg), Celadonite (Cel), Daphnite-14Å (Daph), Diaspore (Dsp), Diopside (Di), Greenalite (Gre), Hydroxyapatite (Hap), Mesolite (Mes), Minnesotaite (Mns), Quartz (Qtz), Saponite-Mg (Sap), Tremolite (Tr). Note that models produced pure C and Fe phases; these may well be amorphous phases rich in those elements, or poorly modeled secondary silicates, oxyhydroxides, or salts.

Water–rock models based on Máaz and Séítah rock unit inputs yielded markedly different results. Figure 3 shows that minerals produced in abundances greater than 0.25 moles (at *ξ* = 1) were common to most models; minnesotaite (a pyrophyllite-talc group mineral), “diopside”, mesolite, and quartz were common. Antigorite and greenalite (two serpentines) were absent from Máaz models, but present in Séítah models. Minnesotaite was absent from Séítah model products. 

In the Máaz models, minnesotaite was the dominant mineral produced, in terms of mass. At equilibrium, the minerals minnesotaite (~0.741 to ~1.06 moles), mesolite (~0.21 to ~0.877 moles), “chromite” (~0.066 to ~0.073 moles) and hydroxylapatite (~0.116 to ~0.133 moles) were produced in all simulations. Annite (~0.154 to ~0.263 moles) and andradite (~0.0079 to ~0.044 moles) were present in all simulations, except in the CaMg(ClO_4_)_2_ case. “Tephroite” was present (~0.033 to ~0.036 moles), except in the CaMg(ClO_4_)_2_ model; annite was produced, except in the CaMg(ClO_4_)_2_ case. Quartz (~0.421 to ~1.44 moles) was produced in all simulations except the Rosy Red and NaClO_4_. “Diopside” (~0.479 to ~0.631 moles) was produced in all models except CaMg(ClO_4_)_2_ and MgNa_2_(ClO_4_)_2_. “Tremolite” (~0.017 to 0.176 moles) was produced in only the MgNa_2_(ClO_4_)_2_ and Rosy Red models. Saponite-Na (~0.024 moles) was produced only in the MgNa_2_(ClO_4_)_2_ models. Pyrite (~0.00011 to ~0.005 mole) was produced in the CaMg(ClO_4_)_2_ and MgNa_2_(ClO_4_)_2_ models. Alabandite (~0.0007 to ~0.00098 mole), a rare Mn-sulfide mineral, was produced in the Ca(ClO_4_)_2_, CaNa_2_(ClO_4_)_2_, and Mg(ClO_4_)_2_ reaction simulations. Albite (~0.158 to ~0.281 moles) was produced in MgNa_2_(ClO_4_)_2_, NaClO_4_, and Rosy Red simulations. Nontronite-Na (~0.0039 mole) was produced only in the CaMg(ClO_4_)_2_ case. Saponite-Na (~0.024 moles) was only present in the MgNa_2_(ClO_4_)_2_ case. Saponite-mg (~0.295 moles), muscovite (0.210 moles), and diaspore (~0.317 moles) were produced only in the CaMg(ClO_4_)_2_ case. “Hedenbergite” (~0.079 to ~0.081 moles) was only present in the Ca(ClO_4_)_2_, CaNa_2_(ClO_4_)_2_, and Mg(ClO_4_)_2_ cases. 

The Séítah simulations yielded greenalite as the mineral in the highest molar abundances (~0.980 to 1.207 moles). In all models, andradite, antigorite, “chromite”, daphnite-14Å, greenalite, hydroxylapatite, and “tephroite” are produced. Molar distributions were: andradite (~0.06 to ~0.179 moles); antigorite (~0.104 to ~0.111 moles); “chromite” (~0.020 to ~0.022 moles); daphnite-14Å (~0.150 to ~0.238 moles); hydroxylapatite (~0.020 to ~0.022 moles); and “tephroite” (~0.051 to ~0.055 moles). Other minerals were commonly produced in Séítah models, but in different proportions. Annite populated nearly all model outputs (~0.010 to ~0.027 mole), but was absent in the NaClO_4_ case. Troilite (~0.001 mole) and “diopside” (~0.119 to ~0.224 moles) were produced in all models except the NaClO_4_ and the Rosy Red cases. Some minerals were only produced in one simulation for the Séítah models: natrolite (~0.076 moles) was only produced in the NaClO_4_ case, phlogopite was produced in the NaClO_4_ models (~0.036 moles), and saponite-Na (~0.145 moles) was produced in only the Rosy Red simulations. 

### 3.2. Statistical Analysis of Simulation Results

**Figure 4:**
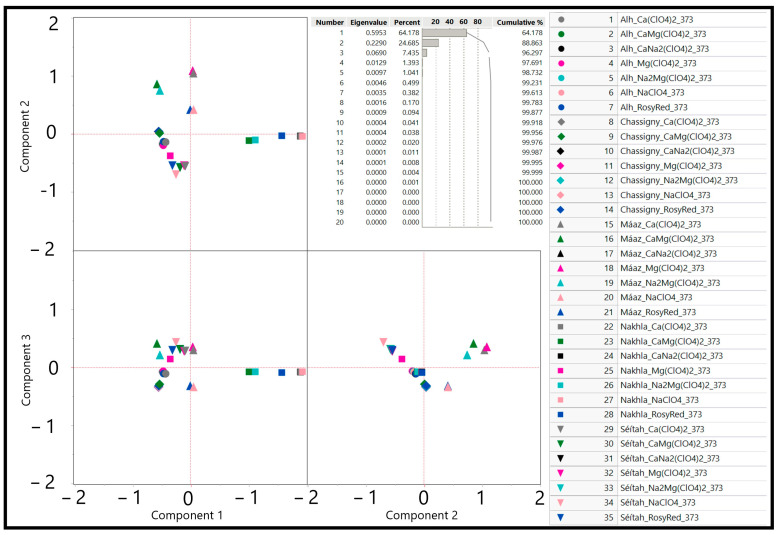
Robust principal component analysis of equilibrium molar abundances of minerals produced. Symbol shape indicates protolith: circles, ALH77005; diamonds, Chassigny; squares, Nakhla; triangle pointing up, Máaz; triangle pointing down, Séítah. Colors of symbols reflect water type. Scree plot shows eigenvalues and percentage of eigenvector influence. See embedded legend. Eigenvalues are based on data covariances.

Robust principal component analysis (RPCA) on covariances was completed and spectral decomposition determined the eigenvalues of the equilibrium modeling of the mineral data. The impact of extreme values was effectively damped, as recommended by Zuo (2013). RPCA results show that the first three principal components, PC1, PC2, and PC3, together account for greater than 95% of the variance in the modeled dataset (Figure 4). Eigenvalues based on covariances for the mineral groups are driven largely by PC1 (>64.17% of the variance), driven by positive loadings of “diopside” (~0.991), “tremolite” (~0.676), and, to a lesser extent, greenalite (~0.498) and prehnite (~0.429). The negative loadings in PC1 are the minerals of antigorite (~−0.661), daphnite-14A (~−0.434), “chromite” (~−0.427), and magnetite (~−0.370). 

PC2 (~24.68% of the variance) is largely controlled by the positive loadings from the minerals of mesolite (~0.874), minnesotaite (~0.874), quartz (~0.811), and hydroxylapatite (~0.806). Negative loadings on PC2 were greenalite (~−0.771), daphnite-14A (~−0.649), andradite (~−0.579), and antigorite (~−0.578). PC3 (~7.44% of the variance) is largely controlled by quartz (~0.519) and magnetite (~−0.628). 

The score plot conveying the interactions of PC1 and PC2 (Figure 4) shows four clusters in the first, second, third, and fourth quadrants. In the first-quadrant–second-quadrant border, the four simulations are the Máaz protolith simulations with Ca(ClO_4_)_2_, Mg(ClO_4_)_2_, NaClO_4_, and Rosy Red. The second cluster clearly in the second quadrant includes Máaz protolith simulations with CaMg(ClO_4_)_2_ and Na_2_Mg(ClO_4_)_2._ The third cluster in PC1 and PC2 includes the majority of the simulations, all of the Chassigny, ALH77005, and Séítah protoliths, across all water types. The fourth cluster in PC1 and PC2 is composed of the Nakhla protolith simulations. Strong geochemical control of the Máaz and Nakhla protoliths is supported. Overall, there is greater dispersion in the first and third clusters when compared to the second.

### 3.3. Bioenergetics

**Figure 5:**
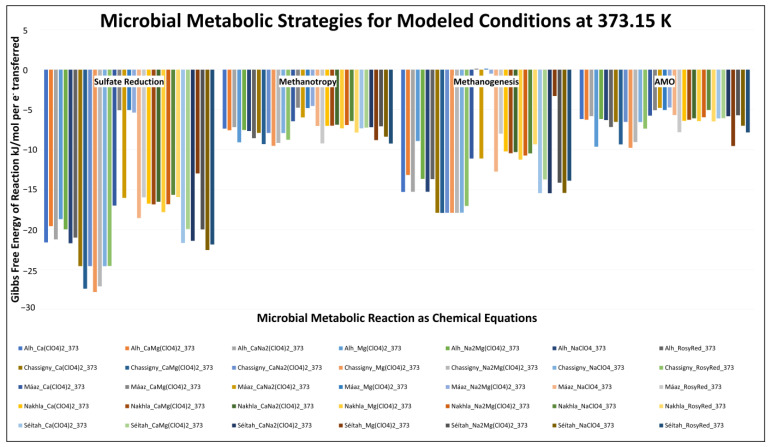
Bioenergetic results from the modeled simulations showing the feasibility of sulfate reduction, methanotrophy, methanogenesis, and anaerobic methane oxidation (AMO), based on Gibbs Energy quantification. All reactions at equilibrium are exergonic at all modeled conditions. Computed Gibbs Free Energy of Reaction is displayed as kJ/mol per e^−^ transferred in the given reaction.

We considered the metabolic reactions for sulfate reduction, methanotrophy, methanogenesis, and anaerobic methane oxidation (AMO), determining each of these metabolic pathways’ theoretical bioenergetic energy yield. Modeled activity values were extracted from Geochemist’s Workbench REACT modeling output files, and used to calculate the thermodynamic feasibility of these reactions, to connect trace-mineral occurrences to pore-space/fracture-network habitability (see Supplementary Table S4 for relevant bioenergetic-species activities). The reactions modeled were nearly all energetically favorable (Figure 5), apart from two reaction models for methanogenesis, although important geochemical triggers vary across models. These two reaction paths, both Máaz protoliths with CaMg(ClO_4_)_2_ and Mg(ClO_4_)_2_ for methanogenesis, exhibited near-zero Gibbs Free Energy values. Relatively subtle changes in the system geochemistry or environmental parameters could shift methanogenesis into a thermodynamically spontaneous space. In other words, upticks in carbon dioxide and hydrogen concentrations or acidity, or sufficient drawdowns in reaction products, could cause these reactions to be energy-yielding. Conversely, the other metabolic reactions are largely exergonic, with sulfate reduction (~−40 to ~−222 kJ/mol), methanogenesis (apart from the two mentioned above) (~−4 to ~−143 kJ/mol), and AMO (~−37 to ~−78 kJ/mol) all considered favorable, under the modeled conditions.

For sulfate reduction, all meteorite protolith-based reaction path models created promising conditions, and were more favorable for sulfate reduction (Gibbs Free Energy values between ~−125 and ~−222 kJ/mol), than were the Máaz and Séítah models (values between ~−40 and −180 kJ/mol). The greatest bioenergetic yield, herein defined as the more thermodynamically favorable negative Δ*G_r_* value for the modeled reaction, resulted from the Chassigny model with the Mg(ClO_4_)_2_-type water (~−222 kJ/mol); the lowest yield resulted from the Máaz model with the Mg(ClO_4_)_2_-type water (~−40 kJ/mol). With respect to sulfate reduction, a general bioenergetic ladder can be constructed as a function of protolith type. From most-to-least favorable for most waters, the protolith types accompanying sulfate reduction would be the following: Chassigny > Séítah > ALH 77005 > Máaz > Nakhla. The exception to this general order was the Séítah reaction with Mg(ClO_4_)_2_. Specifically, for the three meteorite protoliths, Δ*G_r_* values for sulfate reduction are sequentially less energy yielding when arranged in terms of protolith type: ~−222 to ~−196 kJ/mol; ~−173 to ~−149 kJ/mol; and ~−142 to −125 kJ/mol. For the Séítah and Máaz models, the calculated Gibbs Free Energy values for Séítah were greater than all of the respective water reactions with Máaz. There were also differences in the rankings of each specific order of favorability of waters reacting with the protoliths, with only Máaz and ALH 77005 being in the same order. For the Séítah and Máaz models, the calculated Gibbs Free Energy values were similar, but less regular: ~−136 to ~−173 kJ/mol for Ca(ClO_4_)_2_; ~−40 to ~−159 kJ/mol for CaMg(ClO_4_)_2_; ~−128 to −171 kJ/mol for CaNa_2_(ClO_4_)_2_; ~−40 to ~−103 kJ/mol for Mg(ClO_4_)_2_; ~−43 to −159 kJ/mol for MgNa_2_(ClO_4_)_2_; and ~−148 to ~−180 kJ/mol for NaClO_4_. There was a large range in the values, of ~−127 to ~−175 kJ/mol, for Rosy Red.

In our model, methanotrophy was favored across all reaction paths, with energy values between −77 and −36 kJ/mol. The greatest bioenergetic yield resulted from the Chassigny model with the Mg(ClO_4_)_2_-type water (~−76 kJ/mol); the lowest yield resulted from the Máaz model with the MgNa_2_(ClO_4_)_2_-type water (~−36 kJ/mol). The Chassigny models were greater than the other meteorite protoliths for the same water type, with ALH 77005 greater than Nakhla. There was diversity within the protolith reactions, with similarities in most energetically favorable waters, with Mg(ClO_4_)_2_ for Chassigny and ALH 77005. Séítah, Máaz, and Nakhla all exhibited the highest methanotrophic bioenergetic yield with the Rosy Red water. For Nakhla, the range of energy yields was the least among the water types (~−11 kJ/mol), with Chassigny (~−12 kJ/mol), ALH 77005 (~−15 kJ/mol), and Séítah (~−17 kJ/mol) having less range than Máaz-based reactions, which showed the largest range (~−37 kJ/mol). Within methanotrophy only, the Rosy Red waters were the highest-yielding waters for most of the protoliths. 

Regarding methanogenesis, the meteorite-based models were generally more energetically favorable (ΔG_r_ values between −112 kJ/mol and −165 kJ/mol) than the Séítah- and Máaz-based models. Generally, protolith type controlled the feasibility of methanogenesis as calculated here. The greatest energy yield was from the Chassigny model (between −144 and −136 kJ/mol), followed by the Séítah site and ALH 77005, in all reactions except Mg(ClO_4_)_2_, between −124 and −105 kJ/mol. The ALH 77005 and Séítah reactions with Mg(ClO_4_)_2_ were ~−71 kJ/mol and ~−26 kJ/mol, respectively. The Nakhla meteorite models yielded between −90 kJ/mol and −74 kJ/mol. For the Máaz-site models, the reaction path with NaClO_4_ water was the most energetically favorable, at ~−102 kJ/mol, while the reaction with CaMg(ClO_4_)_2_ water was the least favored. In fact, near-zero but positive (not favorable) ΔG_r_ values were calculated for the lowest energy yield resulting from the Máaz reaction path with CaMg(ClO_4_)_2_- and Mg(ClO_4_)_2_-type waters (~1 kJ/mol).

Anaerobic methane oxidation (AMO) was thermodynamically favored in all of the models, generally with negative values of tens of kJ/mol; the greatest bioenergetic yield was for the Chassigny model reacting with the Mg(ClO_4_)_2_ water type (~−78 kJ/mol) and the lowest was for the Máaz-site model reacting with MgNa_2_(ClO_4_)_2_ water type (~−37 kJ/mol). Models following this were (i) Chassigny, ALH 77005, and Séítah, reacting with Mg(ClO_4_)_2_ water, and (ii) Chassigny reacting with CaMg(ClO_4_)_2_ waters and MgNa_2_(ClO_4_)_2_, which had values between −79 and −72 kJ/mol; all others resulted in ΔG_r_ values between −53 and −37 kJ/mol. Séítah, Máaz, and Chassigny models reacted with Rosy Red water to generate geochemical conditions producing ΔG_r_ values of between −63 and −59 kJ/mol. Lower ΔG_r_ values were calculated for Chassigny models with NaClO_4_, CaNa_2_(ClO_4_)_2_, and Ca(ClO_4_)_2_ waters, and also for Nakhla models with Rosy Red, Mg(ClO_4_)_2_, and Ca(ClO_4_)_2_ waters. The least-favorable ΔG_r_ values were calculated for AMO in the Máaz models, when reacting with CaMg(ClO_4_)_2_, Mg(ClO_4_)_2_, CaNa_2_(ClO_4_)_2_, and MgNa_2_(ClO_4_)_2_ waters, and also for Nakhla models reacting with NaClO_4_. These hovered between −41 and −37 kJ/mol. 

## 4. Discussion

Geochemical model results (Figure 2) and subsequent bioenergetics calculations (Figure 5) support multiple fundamentally habitable geologically recent paleoenvironments on Mars, capable of leaving mineral signatures durable enough for long-term detection. To aid comparisons, many primary and secondary minerals detected on Mars in the published literature are provided in Supplementary Table S5; all minerals represented in new model outputs are listed in Supplementary Table S6. As described below in detail, most modeled mineral products are known to occur on Mars, and have been reported previously [157]; the exceptions are andradite, annite, celadonite, and alabandite. Given this reality check, the carbonates, oxides, hydroxides, sulfates, sulfides, and diverse secondary silicates described below are feasible in the Martian shallow subsurface. 

### 4.1. Indicator Minerals of Past Serpentinization (ALH, Chassigny, Nakhla, Séítah)

Serpentine group minerals have been identified on Mars, in layered outcrops, knobby terrain, carbonate plains, the Nili Fossae floor, the wall of the Nili Fossae main floor, impact craters, the Syrtis Major lava flow, heterogeneous terrain, and mélange terrains [47,52]. Recent geochemical and mineral observations at Jezero Crater by the Mars 2020 Perseverance rover show that Séítah formation rock units (with the Dourbes rock sample classed as an ultramafic wehrlite) contain olivine (~65 vol. %), pyroxene (~13 vol. %), and secondary minerals (~12 vol. %) [29]. Further, alteration evidence at these sites includes silicate minerals, Fe-Mg carbonate, and Fe-Mg-Ca sulfate [28]. Given recent discussions of methane on Mars [157,158,159,160,161,162,163,164,165,166], and the potential detections of methane from the Nili Fossae in particular [159,160], altered/altering serpentinites and prospective methane fluxes remain of high scientific interest. Mars-wide serpentine distribution data [50,161,162] set the stage for the investigation of higher-resolution phyllosilicate-bounded permeability networks with fracture-zone habitat and (possibly) channelized transfer of methane from depth to the planetary surface.

Modeling and experimental and analog investigations of low-temperature rock alteration applied to Martian environments yield similar results to the mineral assemblages produced here (e.g., [6,64,66,76,86,87,89,129,163,164,165,166,167,168,169,170,171]). For example, Griffith and Shock [87] modeled the reaction of Icelandic igneous rocks with CO_2_-charged water as an analog for Martian meteorite alteration, using the EQ3/6 software package [version 7]; temperatures were elevated compared to the current study, by 50 °C (the lowest temperature modeled was 423.15 K), yet produced similar minerals—antigorite, annite, phlogopite, daphnite, prehnite, tremolite, andradite, calcite, diopside, hedenbergite, magnetite, albite, quartz, and troilite [87]. Bridges et al. [172] reviewed SNC meteorite mineral assemblages and documented clay minerals in Nakhla, and suggest magnesite, calcite, and the gypsum in Chassigny. Berger et al. [173] used EQ3/6 [version 3245, database R54] to react basalt with a CaCl_2_ brine, and produced similar minerals to this study—Fe, Mg-clay minerals, zeolites, pyroxene, amphibole, epidote and garnet, at 373.15 K. The modeling produced by Berger et al. [173] suggests that the pyroxene and amphibole phases are kinetically inhibited. Catalano [174] performed alteration modeling of Martian basalt at 373.15 K also, using an older version of the Geochemist’s Workbench (ver. 9.0.1) and the Lawrence Livermore National Laboratory thermochemical database V8 R6. Their initial conditions were quite different, focusing on Martian basalts reacting with three dilute H_2_SO_4_ waters (10^−2.5^ to 10^−1^ m) under CO_2_ partial pressures of (*p*CO_2_ between 10^−8^ and 10^−1^ bar). Although these simulations were different water–rock reactions, the simulations yielded similar mineralogical products to our results, notably Fe/Mg saponite, serpentine, and zeolites.

Despite inherent limitations in any database, these diverse models, employing different modeling platforms and selecting different input model components, approximate the same results and correspond to Mars surface observations. Common products across the models are antigorite, cronstedtite-7Å, and greenalite (all serpentine-group minerals). Smectite-group clays such as saponite-Ca, -K, -Na, and nontronite-Ca have been identified on Mars [175,176,177], as have talc [50,64] and chlorite-group minerals [107,175,178,179,180]. Daphnite-14Å (a chlorite), and a diversity of minor minerals that may not be spectrally resolvable (e.g., fine, dispersed metals, such as sphalerite, see Table 1), are also predicted to occur. Spectrally identified serpentine- and smectite-group clays, along with olivine, at/near the Nili Fossae carbonate plains correspond well with our results (e.g., CRISM observations FRT000028BA_07 and FRT0000A09C_07, [50]). Specifically, Amador et al. [50] describe serpentine- and smectite-group clay detections that correspond with modeled results for (a) Nakhla reactions with Mg(ClO_4_)_2_ and NaClO_4_ waters, (b) ALH77005 reactions with NaClO_4_ and Rosy Red, and (c) Séítah reactions with Rosy Red.

A new interpretation of the presence/absence of minor minerals, arguably diagnostic for the specific rock–water reaction paths modeled here, suggests geologically recent paleowater geochemistry and geologically recent-past microbial metabolic opportunity. All protoliths had at least two scenarios where indicator mineral groups were clear (Table 1).

- For ALH77005 models, trace saponite-Na was produced in NaClO_4_ and Rosy Red models.
- Chassigny models produced “Fe” and magnetite for Ca(ClO_4_)_2_, Mg(ClO_4_)_2_, MgNa_2_(ClO_4_)_2_, CaNa_2_(ClO_4_)_2_, and CaMg(ClO_4_)_2_ waters, and produced “Fe” and phlogopite in NaClO_4_ and Rosy Red solutions.
- Nakhla models yielded “tremolite” in all reactions except the Rosy Red, which uniquely produced only minnesotaite; additionally, saponite-Ca was produced in the Mg(ClO_4_)_2_ model, saponite-Na was produced in the NaClO_4_ model, and minnesotaite with prehnite accompanied tremolite in the Ca(ClO_4_)_2_ model.
- In Séítah models, the minerals produced in the NaClO_4_ model were natrolite and phlogopite; the Rosy Red solution uniquely produced saponite-Na.

In general, minor minerals in modeling outputs have been identified/discussed in the Mars-relevant surface mineralogy literature (cf. [179]). In this context, mesolite (zeolite group) has been discussed [133], and populates model results. Tremolite, a common amphibole-family phase in the model results in this work, relates to inferred phases on Mars, such as mixtures of smectites and hornblende [177], and there have been amphibole phases (assumed to be primary) identified in the Tissint Martian meteorite [181]. Al-smectites are reasonable amphibole-weathering products [178]. Additionally, Mars observations of sulfur-bearing phases ranging from gypsum [182] to pyrite [183], and iron-rich phases such as hematite [184,185,186,187] and magnetite [188], are consistent with model results.

### 4.2. Indicator Minerals for Mafic Unit Alteration (Máaz)

Basalt has been identified on the Martian surface via orbital spectroscopic data (e.g., [189,190,191,192]) and rovers [27,28,29,70,125,188,193,194,195,196,197,198,199,200,201,202,203,204]. The Mars meteorite record includes mafic rocks such as basaltic shergottites, gabbroic shergottites, and many nakhlites (e.g., [21,125,205,206]). 

Basalts on Mars range in age and degree of alteration [204], and significant weathering of basalts is implied by many Mars surface observations [207,208]. Mars locations display hematite [209], gypsum [210,211,212,213] carbonate and perchlorates [36,132], and sulfates and silica [204]. Additional secondary minerals such as prehnite, illite/muscovite, serpentine, opaline silica, and the zeolite mineral analcime [175,177,179,214] have been reported. Clay minerals like nontronite [45,215], Mg-smectites [175], aluminum phyllosilicates, kaolinite, beidellite, and montmorillonite are known to occur, with and without nearby hydrated silica [45,216,217]. The Máaz reaction-path models showcase mafic rock alteration as a function of water type, and provide grounds for comparison with existing data for Mars surface mineralogy.

In Máaz models, for all paths, minnesotaite was produced. Quartz was produced in Ca(ClO_4_)_2_, Mg(ClO_4_)_2_, CaNa_2_(ClO_4_)_2_, CaMg(ClO_4_)_2_, and MgNa_2_(ClO_4_)_2_. Co-occurring minerals differed. Ca(ClO_4_)_2_, CaNa_2_(ClO_4_)_2_, and Mg(ClO_4_)_2_ water types all produced minnesotaite and quartz with alabandite and hedenbergite. Both the NaClO_4_ and Rosy Red water types produced minnesotaite with albite and analcime. Both CaMg(ClO_4_)_2_ and MgNa_2_(ClO_4_)_2_ water types reacted to give unique indicator-mineral results. In the CaMg(ClO_4_)_2_ reaction, indicator minerals include nontronite-Na, pyrite, diaspore, muscovite, and saponite-Mg, added to the quartz and minnesotaite background. In the MgNa_2_(ClO_4_)_2_ reaction, albite, saponite-Na, pyrite, and tremolite were present, added to the quartz and minnesotaite background.

The minerals so produced are known in the terrestrial basalt record, and validate the modeling approach. Terrestrial basalts which are altered in marine environments, typically exhibit selective mineral solution and replacement with secondary minerals, over time. Classic processes active in basalt alteration under diagenetic conditions (arguably through the onset of low-grade metamorphism) include albitization or spilitization, zeolitization, and rodingite formation (or rodingitization) (e.g., [218]). 

Albitization/spilitization generally encompasses the transformation of Ca-rich plagioclase to albite at low temperatures, when Na^+^-bearing waters flow through basalts over time. Recall that higher-temperature albitization has been observed for decades in natural environmental samples and experimental data [219], and hydrothermal alteration of terrestrial basalts has conventionally been assigned a mineralogical assemblage of albite-actinolite-chlorite epidote, with quartz and pyrite as possible accessory phases [220]. The modeled increases in albite, chlorite, and quartz certainly correspond to well-known terrestrial basalt alteration. 

Zeolites are hydrous aluminosilicates that have great capacity for ion exchange. Zeolitization has typically been associated with mafic glass alteration or cavity-filling mineralization in vesicular basalt. In fact, seabed coring e.g., [221] has often documented pervasive zeolite formation, with calcite mineralization as a late-stage process. In other locations, mineral zonation observed within the zeolite-group minerals has allowed generations of low-temperature alteration to be resolved. In thickly stacked basalt flows of Iceland and East Greenland, experiencing burial metamorphism in the presence of advecting waters, analcime was detected in subsurface horizons ~600 to 750 m and 500 to 800 m below the top of the basalt pile, with mesolite detected at adjacent, deeper intervals [222]. Zeolite zonation, given distinct temperature gradients, has been highlighted in Icelandic geothermal systems: mesolite has been associated with 343.15 K to 363.15 K alteration temperatures, and analcime with the 503.15 K to 573.15 K interval [222]. Alterations in volcaniclastic sediments in Japan resulted in clinoptilolite and analcime forming in temperature ranges of 333.15 K to 357.15 K and 364.15 K to 393.15 K, respectively [222]. In West Greenland basalts, regionally low geothermal gradients (<45 °C/km) drive the formation of sequential classes of zeolites, with some incorporation of ‘mafic phyllosilicate’ material [223]. Weisenberger and Selbekk [224] documented analcime and mesolite in Iceland’s Hvalfjordur basalt flows, post low-temperature alteration, with robust phase detections. Fresher water systems (continental environments) also likely host zeolite formation, as shown experimentally [225], and increasingly in field observations [222]. 

The case of rodingitization is particular, and geochemically very specific: calcium-rich waters acting on mafic lithologies (Ca-metasomatism), often associated with serpentinization [226], drive the formation of specific low pressure–temperature mineral assemblages including prehnite, tremolite, diopside, hydrogarnet, epidote or clinozoisite, chlorite, calcite, and rare titanite, apatite, and zircon [226,227,228,229,230,231]. Many of these minerals occur in model outputs. The common terrestrial rodingite-mineral features, functionally controlled by the availability of Ca^2+^-bearing terrestrial waters, might necessarily be replaced by Na^+^ or Mg^+^ mineral phases, based on the major cation inventories in the modeled waters.

### 4.3. Bioenergetic Implications

Subsurface serpentinization provides the necessary chemical energy to support microbial metabolisms, both on Earth and beyond ([1,149,232,233,234,235,236,237]; this work). The challenge of approximating extraterrestrial growth conditions has been innovatively approached. Using a Chassigny-type protolith, Ref. [238] showed that it is possible to produce approximately 26 g of biomass using chemical energy derived from water–rock reactions. Here, water–rock mass ratios were defined as nearly 1:1, relatively low ratios, indicating slow or low-volume percolation of reactive water in the system. There are further factors needed to make a particular environment important for astrobiology, such as conducive geophysical conditions like planetary mass and size, stable temperature, protection from galactic cosmic radiation, etc., and star–planet interactions. 

When computed based on activity data in modeling outputs (see Figure 5), we show that the spontaneous reactions are the following: sulfate reduction coupled with H_2_ oxidation; methanogenesis, using CO_2_ as the carbon source to be reduced, coupled with H_2_ oxidation; and anaerobic methane oxidation (to aqueous CO_2_), coupled with sulfate reduction. 

Aqueous alteration of mafic rocks certainly influences the subsurface bioenergetics in terrestrial mafic-rock units, though chemically and energetically distinct from serpentinization processes. Hot, young mafic crusts, with abundant ferrous iron, can facilitate diverse Fe oxidation reactions, but cooler microbial ecosystems may shift to take metabolic advantage of radiolytic hydrogen production as the key electron donor [239]. When olivine-rich lithologies experience low rates of water flow through the permeable matrix, hydrogen is not lost as rapidly, and may host significant hydrogenotrophic activity [240]. The cooler systems are the more apt comparisons here.

The bioenergetic implications of our modeling are that there are high-, moderate-, and low-yield metabolisms, feasible across the water types considered. High-yield metabolisms involve sulfate reduction and methanogenesis (~−100 to −250 kJ/mol). Moderate-yield (between −100 and −50 kJ/mol) and low-yield (>−50 kJ/mol) metabolisms involve a few methanogenesis reactions, along with all methanotrophy and anaerobic methane oxidation (AMO) reactions. 

When methane oxidation (to aqueous CO_2_) is coupled with sulfate reduction (to H_2_S_(aq)_), the ΔGr of methanotrophy is strongly positive (non-spontaneous) in every modeled case, at every condition. Very little variation exists in the overall energetics of the reaction, based on the modeled conditions. Our results are within the range of results obtained by Marlow et al. [235], who investigated seven fluids and calculated methane oxidation using the same summarized metabolic reaction, obtaining values between −31 and −135 kJ/mol. Differences in our calculations likely reflect differences in model structure and the decision to allow planetary fluids or Martian groundwaters to react only with the Martian atmosphere [235], rather than investigating the subsurface water–rock system (this study). 

Note that bioenergetic calculations for reactions discussed here give numerical results that are an order of magnitude less than those reported in Oze and Sharma [20]; we present ΔG_r_ (tuned to activity data in model results), while standard-state (ΔG_r_^◦^) values were discussed conceptually in the work of Oze and Sharma [20]. Model outputs in this study are thermodynamically stable at equilibrium, and mineral assemblages are consistent with other work on the low-temperature alteration of mafic- and ultramafic-phase equilibrium models on Mars [241]. Kinetic considerations are important to real-weathering and low-temperature alteration processes at/near the surface of Mars, but are beyond the scope of this work. Within the paragenesis of water–rock settings with astrobiological implications, such as these, it is important to recognize that, although the temperature is relatively low for geological processes, alteration of primary minerals to secondary phases is possible, for example by protons or catalysts that weaken surface charges and atomic bonds [173]. Further, this process is still being explored kinetically, and an example of this is the transition of smectite to illite, shown in similar quasi-diagenetic alteration conditions [242,243,244]. In addition, some high-temperature minerals, such as fayalite, have been suggested to form far below convention, at 573 K, in a Washington State gold mine [245]. The thermodynamic stability and challenges to conventional kinetic limitations have also been explored in space materials, including carbonaceous chondrite MAC 88107, suggesting that 423 K to 473 K aqueous alteration can produce iron-rich olivine, via precipitation [246]. 

### 4.4. Precedents from Similar Terrestrial Microbial Ecosystems

As Baas Becking stated in 1934—the environment selects for the life it hosts. Chemolithoautotrophic organisms are driven by chemical energy obtained from the Earth’s lithosphere everywhere beyond the reach of sunlight, where temperature and other conditions allow. In continent-hosted serpentinite aquifers, similar water–rock reactions impact subsurface groundwater ecosystems [151,152,247,248,249,250,251,252,253] (Supplementary Table S1). In the Coast Range Ophiolite, USA (e.g., [254,255]), the Samail ophiolite in Oman (e.g., [256,257,258]), the Bay of Islands Ophiolite in Newfoundland, Canada (e.g., [259,260]), the Santa Elena Ophiolite, Costa Rica (e.g., [261]) and others, serpentinization influences biogeochemical cycles tuned to the geochemistry of the environment. Extraterrestrial environments could host similar biotic processes [7,50,54,55,56,71,161,260]. 

Possible sites of serpentinization-driven metabolic systems have also been identified, such as the Alter-do-Chão massif [262] and the Ronda massif [263]. Notably, at the Outokumpu deep drill hole, serpentinization at depths between 1.3 and 1.5 km drives carbon assimilation, methanogenesis, nitrate reduction, and sulfate reduction [264]. This particular site is analogous to sites in the Martian crust and upper lithosphere, where the habitable volume encompasses depths of 6–8 km, before reaching upper temperature limits for life [39]. 

The subsurface seabed (sedimentary blanket and mafic-to-ultramafic rocks of the oceanic lithosphere) supports an array of metabolisms. Where serpentinization occurs in fault-translated or underlying ultramafic units, it generates redox gradients and a long-lived source of carbon (organic acids and methane, reworked from ocean-derived inorganic and organic compounds) and of electrons (generally, but not exclusively, via H_2_ oxidation). Deep sediments support sulfate reduction (e.g., sulfate-reducing methanotrophy, [265,266]), as do vigorous, widespread hydrothermal vent systems [267,268,269,270,271] and paleo-hydrothermal environments, such as Río Tinto in the Iberian pyrite belt [272]. Methane cycling has been closely studied in marine vent systems, such as at the low-temperature Lost City Hydrothermal Field [273,274], and higher-temperature vent systems, like the Rainbow and Logatchev Mid-Atlantic Ridge sites [271]. In more recent studies of microbial colonization of ultramafic materials in a seabed-simulation experiment, special emphasis has been placed on prospects for H_2_ scavenging and its efficient utilization in the seabed [275].

Water–rock interactions fuel the deep biosphere in the terrestrial mafic–ultramafic subsurface, at temperatures most often above 274.65 K to ~310.15 K (e.g., the ophiolite-hosted groundwater systems in Gruppo di Voltri, Liguria, Italy, and Oman; see Schrenk et al. [271]), or in seabed vent systems from 313.15 K up to ~653.15 K [271]. A landmark study, although controversial, of the role microbes play in the cool Columbia River Basalt-hosted aquifer revealed a slow but active influence on dissolved H_2_ abundances (e.g., [276]). 

Life exists in ice, icy-regolith and icy-aqueous settings (oceanic, lacustrine, or pore spaces in sediments), and cold arid deserts [75], and is under close study in permafrost generally [277], with special attention paid to Alaskan permafrost [278]. Further, in cold arctic-lake sediments, terrestrial methanogens such as Methylobacter and Methylomonas show evidence of methane oxidation at extremely low temperatures, down to at least 277.15 K [279]: life persists near the lower-temperature cut-off for survival. Although beyond the scope of this modeled work, the temperature resilience of methanogens and other cryophilic microbes makes them excellent model systems for comparative study, as lower-temperature systems come under scrutiny.

Biogeochemical limits of course constrain life on Earth. In most land-based Earth environments, the rock-hosted shallow biosphere is richly supported by photosynthetic biogeochemical cycles, directly or indirectly, through the decomposition of organic matter produced in sunlit systems (see [75] and references therein, [280,281,282,283,284,285]). Diverse metabolic pathways and complex ecological relationships exist, and understanding the dynamics of subsurface ecosystems is crucial to applying Earth analog datasets elsewhere. Of special interest are microbes that assimilate carbon as CO_2(g)_ or metabolize other relevant Martian atmospheric gases, such as H_2_, CO [286], and CH_4_ [287,288]. 

Microbes carrying out the metabolic reactions tested here include the anaerobic methanotrophic archaea (ANME), for which the mcrA gene was identified at Lost City [289], and methanotrophic bacteria like Methylococcales, also detected at Lost City [271] and at Rainbow [290]. Furthermore, Methanobacterium has been identified at the Zambales [291] and the Del Puerto Ophiolite [292]. Given that methane production is known to occur in serpentinization systems, direct observations of gene sequences associated with methanogenesis have been successful in the Del Puerto Ophiolite [292], the Bay of Islands Ophiolite [250], and at Lost City [273]. Methanogenic archaea have also been observed at the South Chamorro Seamount [292], and archaea in the order Methanococcales were documented at sites in the Mid-Atlantic Ridge, such as at Logatchev and Rainbow. In our bioenergetic models, H^+^, SO_4_^2−^, HCO_3_^−^, S^2−^, and CO_2(aq)_ were usually the limiting reagents. 

In the context of work by Marlow et al. [235], patterns are similar, though Marlow et al. used contrasting fluids and calculated methane oxidation using the same methanotrophy; they summarized the metabolic reaction, and obtained values between −31 and −135 kJ/mol CH_4_. However, a key difference between reports is that Marlow and colleagues allowed Martian groundwaters to react with the Martian atmosphere; we keep the water–rock reaction path closed in the rock subsurface. 

Comparative terrestrial analog studies remain in high demand; terrestrial microbes toggle between different metabolic pathways based on environmental triggers, and there are many plausible dynamic scenarios for different Martian environments. In any case, the absolute habitable volume in the near-subsurface of Mars must vary over space and time. Debate continues about the nature of a shallow-water reservoir on Mars. There may have been ‘sporadic’ groundwater upwellings on local scales [39], near global groundwater systems [41], and modern groundwater at a depth of several km has been posited [293]. Geomorphology supported by landscape models suggests that numerous short-lived Noachian lake basins were exposed at the planetary surface [294]. In a troubling counterpoint, no shallow Martian aquifer was actually detected in InSight seismometer datasets [295], but there is consideration of possible liquid-phase water via observations of reoccurring slope lineae [296,297]. 

An important area of future work will be in evaluating the bioenergetics and biomarkers of acetate-producing microbes such as those recently detected in Oman [298] and which are strongly suspected at The Cedars ultramafic-hosted springs in northern CA [299] and the Hakuba Happo hot springs, with some serpentinization-derived mixing component, in Japan [300]. Given the repeated observations of acetate and other organic acids in waters influenced by serpentinization, the growth limits and preferred aqueous environments of known acetate-generating microbes may have profound importance in extraterrestrial settings. 

### 4.5. Modeled Results in the Context of Planetary Science Missions

Recent ground-penetrating radar measurements collected by the Perseverance rover show both fast and slow travel times, indicating that the near-subsurface is heterogeneous [27]. The non-uniform nature of the near-subsurface adds new relevance to this study. Recall that, for the model platform used here (Geochemist’s Workbench), the system pressure is set for the system at 1.013 bar: this pressure corresponds to shallow, near-subsurface depths (~9 m). Although some minerals/polymorphs were not present in the thermodynamic database utilized in the modeling (e.g., compositionally diverse brucite minerals), we have described sets of minerals that connect to clearly defined water–rock interaction histories. In other words, the mineral groupings presented here constitute a hydrogeochemical facies model that can be consulted, given mineral observations from Mars and other planetary surfaces with mafic–ultramafic exposures.

With the anticipated future retrieval of rocks cored by the Perseverance rover at the Maaz site, core splits should be screened carefully for indicator minerals such as pyrite, alabandite, and zeolites. Regarding pyrite: modeling the Máaz protolith reactions with CaMg(ClO_4_)_2_ and Na_2_Mg(ClO_4_)_2_ generated pyrite. Recall that pyrite has been detected in Martian meteorites ALH 84001 [301] and NWA 7533 [302], and previously on the Martian surface at the Gale crater, in association with clay minerals [183,303]. Regarding alabandite, a rare Mn-sulfide: selected Máaz protolith reactions of Ca(ClO_4_)_2_ and CaNa(ClO_4_)_2_, and the simulations with Mg(ClO_4_)_2_ produced alabandite chemically analogous to pyrrhotite, with diverse cations bonded to sulfur. Regarding zeolites: analcime was produced in the modeling for the models, Na(ClO_4_)_2_, and reactions with Rosy Red co-occurring with smectites, serpentines, magnetite, clinopyroxenes, hematite, and carbonates. Perseverance cores sampled at the Séítah site should be screened for minerals that emerge from ultramafic protolith models, and are diagnostic for specific water types. 

For context, Pascuzzo et al. [304] suggest that the Nili Fossae likely had shallow subsurface water–rock reactions and hydrothermal activity. Both Amador et al. [50] and Viviano et al. [64] describe rock alteration scenarios involving serpentinization followed by carbonation of the olivine-rich unit. Serpentinization likely altered the olivine-rich, uppermost unit at the nearby Nili Fossae [46,50,60,61,79,215], specifically in the olivine-rich caprock, between 35 m and 160 m thick [165]. The prospect of a wedge of habitable, evolving ultramafic/mafic material, providing bioenergetic support for life based on these transparent modeling results, is compelling. In fact, related work on the Nili Fossae suggests fluid-limited carbonation of serpentine at low-temperature (≤200 °C) [64,305,306], immediately relevant to the low-temperature conditions considered in this study.

Another exciting connection with timely investigations related to the Mars 2020 mission rests in the recent detections of organic carbon at specific sites. Recent work by Scheller et al. [307], which analyzed Máaz- and Séítah-formation rocks, identified organic material associated with Na-rich perchlorates in rock units similar to initial and equilibrium assemblages in this study. Interestingly, Scheller et al. [307] noted that serpentine minerals have not been identified yet in the Séítah-formation rocks, yet these remain ultramafic in a geochemical sense; a serpentine phase or a post-serpentine weathering phase (complexly interlayered chlorite–smectite assemblages) would be consistent with our understanding of mineral evolution in these systems. 

As an aside, detections of organic molecules in diverse Mars materials are mounting: the Mars meteorite ALH 84001 exhibits organic molecules associated with serpentinization [308], building on much previous work [205,309,310,311,312,313,314,315]. Even more profound is the organic-molecule detection in the approximately 3.5 Ga mudstones at Pahrump Hills, Gale crater, by the Curiosity rover [316], and in the atmosphere [147]. 

Mars hosts diverse minerals related to mafic–ultramafic rocks and their alteration, which have been observed either through rover tools or through remote sensing, and to which the scientific community will shortly have access. At least 160 distinct mineral species have been identified on, or are postulated to be on, Mars [155], Detailed micro- and nano-scale mineralogical study of returned Mars samples after the Mars 2020 mission, ongoing investigations of the Jezero Crater, or future high-res sampling in/around the Nili Fossae region will benefit from targeted modeling results. Key indicator minerals are established for reference in future Mars mission-data reduction, to confirm geologically recent habitability in sampled rocks and related environments on Mars, and to direct follow-on analytical work focused on biomarker detection.

### 4.6. Modeled Results in the Context of Geologically Recent Habitable Niches 

Terrestrial-planet volcanism and impact events drive hydrothermal activity, and the subsequent water–rock interactions have the potential to drive microbial ecosystems (e.g., [1,2,3,4,5,6,7,96]). Magmatic and impact-induced hydrothermal activity was periodic throughout the geological history of Mars (e.g., [316] and references therein). Volcanism and impact events can create spatially limited but important geophysical conditions that are sufficient for driving water–rock reactions in microenvironments pertinent to this study. 

Scattered throughout the Tharsis region of Mars, evidence of volcanic activity younger than 100 Ma is found the southeast approach to Olympus Mons, the northeast approach to the Ceraunius Fossae, the southeastern approaches to Ascraeus Mons and Pavonis Mons, and the northeast approach to Arsia Mons [317]. New evidence suggests that the Tharsis region on Mars, specifically Alba Mons, has been active continuously, as recently as ~10 Ma, with a large number of ~100 Ma-aged eruptions suspected between 20 and 25° N and 125 and 105° W associated with Alba Mons and the Ceraunius Fossae region [318]. Krishnan and Kumar [318] suggest that the recent tectonic and volcanic activity drove the boulder falls hypothesized to be the epicenter of the observed M_W_ 4.1 marsquake on 18 September 2021. Serpentine and associated minerals in the Tharsis Montes have been strongly inferred spectrally, but require further characterization [319]. 

Impact events are geologically pervasive, and can generate significant heat, which will result in the planetary lithosphere having an increase in temperature [320]. Small-impact cratering events, between 5 and 10 km, have the potential to drive hydrothermal systems on Mars [96]. A moderate 30 km-diameter crater on Mars has the potential to fuel a hydrothermal system in the subsurface for ~67,000 years [321]. Further, analysis of analog impact-induced hydrothermal systems found on Earth, such as the relatively small ~4 km-diameter crater at Kärdla, Estonia took between ~1500 and 4000 years to cool to below our model temperature of 373.15 K [322]. Larger, less-common impact events studied on Earth, such as the ~250 km-diameter Sudbury impact event is likely to have sustained hydrothermal activity for approximately 1 million years [323]. Modeling work associated with the Chicxulub impact, a 180-km-diameter event, suggests 1.5 to 2.3 million years [324]. 

Impact-induced plausible hydrothermal systems have been charted in diverse terrains on Mars. For context, ancient examples include the Noachian ~153 km-diameter Holden impact crater in the Margaritia Sinus quadrangle [325] and the ~40 km-diameter Hesperian Toro complex impact crater in the Sytris Major quadrangle [326]. Within the geologically recent Amazonian, two craters have been identified that have likely hosted hydrothermal systems; spectral detections of phyllosilicates support this [327] at an unnamed ~20 km-diameter crater in the Ismenius Lacus quadrangle (from [328]: Crater ID 05-000375), and at the ~62 km-diameter Stokes impact crater in the Cerbrenia quadrangle (from [328]: Crater ID 07-0000008). The Ismenius Lacus crater shows spectral evidence of chlorite and Fe-serpentine, where the Stokes impact crater shows spectral evidence of a suite of Fe/Mg/Al phyllosilicates [327]. 

Given these two impact craters with spectrally defined hydrothermal phyllosilicates, along with appropriate impact-event causes (e.g., no hydrothermalism suspected prior to impact), Turner et al. [327] identified 144 craters ≥ 7 km in diameter within the Amazonian. Turner et al. [327] further examined craters with diameters of 3 to 7 km, and found that these craters lacked hydrated minerals in the CRISM data. Further, Turner et al. [327] described hydrothermal systems during the Amazonian period on Mars as sparse processes, not easily detectable at the spectral spatial scale of ~20 to 40 m/pixel resolution of the satellite observations by CRISM. Others have hypothesized that the physicochemical conditions for water–rock reactions associated with impact events could drive habitable environments for extended periods of time [321,329,330]. During the Amazonian, impact-event frequency has been much lower than during the Noachian or Hesperian, but [331,332] indicate that crater diameters between 8 and 64 km, relevant to this study, would occur on the surface of Mars every ~1 million years (in the 8 km-diameter case) or ~100 million years (in the 64 km-diameter case). Smaller craters, such as those with ~1 to 2 km diameters, could occur as frequently as every ~10,000 years (~1 km-diameter case) to ~100,000 years (~2 km-diameter case), but such craters have yet to be identified with hydrothermal mineral assemblages on Mars [327]. 

Radionuclides have also been considered as a heat source to plausibly spur hydrothermal systems [2,4,331] and subsurface microbial metabolisms in the modern Martian surface (e.g., [76]). Radionuclides, such as U, Th, and K (half-lives in the order of ~ billions of years) have the capacity to drive hydrothermal systems for timescales significantly greater than volcanism and impact events, in the order of >100 million years [331]. Examination of the Martian near-subsurface for areas of high concentrations of Th and K by Ojha et al. [331], showcased the Eridania quadrangle region and Isidis Planitia just southeast of the Nili Fossae, in the Syrtis Major quadrangle. Both sites exhibit spectral evidence of serpentinization via the identification of Fe- and Mg-serpentine and other products [50,332]. Relevant investigation continues also at an analog site on Earth for radiogenic heat-derived hydrothermal systems at the Mt. Gee-Mt. Painter system in the Northern Flinders region of South Australia (MGPS) [331].

Magmatic activity, impacts, and radiogenic heat production are considered the three planetary-scale processes that generate the heat necessary for the water–rock modeling in this study. Temperatures at/near 373 K occur at diagenetic/very low-grade metamorphic conditions [12,13,64,305], and are certainly habitable for life as we know it [39]. Although outside the scope of this study, modeling lower temperatures, between 273.15 K and the temperature limit used in this work, would provide further insight into the thermodynamic stability of phases which may or may not be kinetically limited in the Martian subsurface environment. We look forward to further interrogation of plausible hydrothermal sites and serpentinization in planetary environments, leveraging the fine-tuning (particularly in terms of solid-phase and aqueous-system geochemistry) possible with a thermodynamic modeling approach. 

## 5. Conclusions

The surface and near-surface of contemporary Mars is frozen, oxidized, and irradiated. However, in the past, Mars likely had habitable conditions with the potential to sustain life [333,334,335,336,337,338], and may still today hold habitable niches in the subsurface, supported by water–rock reactions. Between 3.5 and 3 billion years ago in Martian history, encompassing the middle-to-late Noachian Era and the early Hesperian Era [334,335], there is evidence that the planet was geologically much more active, and had liquid water (e.g., [134,296,334,335,336]), but evidence of modern groundwater flows (e.g., [339,340,341,342,343,344]) excites prospects into a rock-hosted habitable environment within the subsurface of Mars (this work, [76]). Our work shows that the chemical energy available through aqueous alteration of specific units in the Martian lithosphere corresponds to enhanced potential for microbial metabolism, charting a possible pathway for habitability under near-surface geophysical water–rock conditions, at plausible temperatures for proximal geothermal or impact-derived heat. We show that reactions that mirror the known terrestrial microbial metabolisms of sulfate reduction, methanogenesis, methanotrophy, and AMO are spontaneous, from a thermodynamic perspective, and are feasible metabolic strategies for the modeled environment.

A key takeaway of this work is that our models confirm that mineral detections on Mars by the Compact Reconnaissance Imaging Spectrometer for Mars (CRISM) in the Nili Fossae region [46,50] can be produced through past water/rock interactions under geologically recent Martian conditions, with specific water types implicated by minor mineral occurrences. The rocks under study inform ongoing investigations by the Perseverance rover. The water types used to structure the modeled interactions are literature-based, and feature different cation–perchlorate compositions. The overlap between modeled mineral products and the growing lists of minerals detected on Mars is now better contextualized. 

Beyond the current Mars mission, other locations on Mars where there is likely serpentine and other minerals predicted in this study include the Claritas Rise, Valles Marineris, North Argyre Basin, Tyrhenna Terra, South Isidis, Nili Fossae, and Terra Sirenum [46,49,50,337,338,339,340,341,342,343,344,345].

Table 1 provides a condensed summary of indicator minerals that derive from specific water/rock interactions under Martian conditions, and can be used as a reference for screening well-resolved mineralogical data for these ‘fingerprints’ of geologically recent paleo water activity. Applying these results to the analysis of Mars samples (in situ and when returned to formal laboratory settings on Earth) in future NASA missions is exciting.

Future directions include the incorporation of other Martian crustal compositions with explicit influx of radiolytic hydrogen, and consideration of other possible water types. Better representation of the Martian near-surface environment, and systematic assessment of mineral suppression choices with metabolic considerations, would deepen the model result (cf. [4] on icy satellites). Continued monitoring of terrestrial analog geomicrobiology of continental and submarine serpentinites, basaltic aquifers, and the dynamics and resilience of metabolic pathways used by these organisms on Earth would also help constrain how far we can take modeling in assessing the astrobiological potential of the Martian near-subsurface and similar environments elsewhere. The importance of serpentinization as a spur for life and a sustainer of astrobiology may be far greater than previously assumed.

## Figures and Tables

**Table 1:** Indicator minerals of specific water–rock reaction paths, modeled at 373.15 K. Not all reaction paths produced diagnostic mineral groups. Key indicator minerals were saponite-Na, minnesotaite, “tremolite”, and zeolite-group minerals.

Protolith	Water	Indicator Minerals Present at Equilibrium
ALH77005	NaClO_4_	Saponite-Na
	Rosy Red	Saponite-Na
Chassigny	Ca(ClO_4_)_2_	“Fe”, Magnetite
	CaMg(ClO_4_)_2_	“Fe”, Magnetite
	CaNa_2_(ClO_4_)_2_	“Fe”, Magnetite
	Mg(ClO_4_)_2_	“Fe”, Magnetite
	MgNa_2_(ClO_4_)_2_	“Fe”, Magnetite
	NaClO_4_	“Fe”, Phlogopite
	Rosy Red	“Fe”, Phlogopite
Nakhla	Ca(ClO_4_)_2_	Minnesotaite, Prehnite, “Tremolite”
	CaMg(ClO_4_)_2_	“Tremolite”
	CaNa_2_(ClO_4_)_2_	“Tremolite”
	Mg(ClO_4_)_2_	Saponite-Ca, “Tremolite”
	MgNa_2_(ClO_4_)_2_	“Tremolite”
	NaClO_4_	Saponite-Na, “Tremolite”
	Rosy Red	Minnesotaite
Máaz	Ca(ClO_4_)_2_	Minnesotaite, Quartz, Alabandite, “Hedenbergite”
	CaMg(ClO_4_)_2_	Minnesotaite, Quartz, Nontronite-Na, Pyrite, Diaspore, Muscovite, Saponite-Mg
	CaNa_2_(ClO_4_)_2_	Minnesotaite, Quartz, Alabandite, “Hedenbergite”
	Mg(ClO_4_)_2_	Minnesotaite, Quartz, Alabandite, “Hedenbergite”
	MgNa_2_(ClO_4_)_2_	Albite, Minnesotaite, Quartz, Saponite-Na, Pyrite, “Tremolite”
	NaClO_4_	Albite, Minnesotaite, Analcime
	Rosy Red	Albite, Minnesotaite, Analcime
Séítah	NaClO_4_	Natrolite, Phlogopite
	Rosy Red	Saponite-Na

## Data Availability

All data are available in Supplementary Files.

## Supplementary Materials

The following are available online at https://www.mdpi.com/article/10.3390/life13122349/s1, Table S1: Serpentinization Sites, Table S2: Protoliths, Table S3: Aqueous Species, Table S4: Aqueous Species Activities, Table S5: Mineral Products, Table S6: All minerals represented in new model outputs, Figure S1: Model pH.
