# Supplementary material for: Mineral Indicators of Geologically Recent Past Habitability on Mars
Source: Life (Basel). 2023 Dec 15;13(12):2349. doi: 10.3390/life13122349 (PMC10744562; doi:10.3390/life13122349)
Supplement: Supplementary file 1 [file life-13-02349-s001.zip › Supplemental Figure 1.pdf]

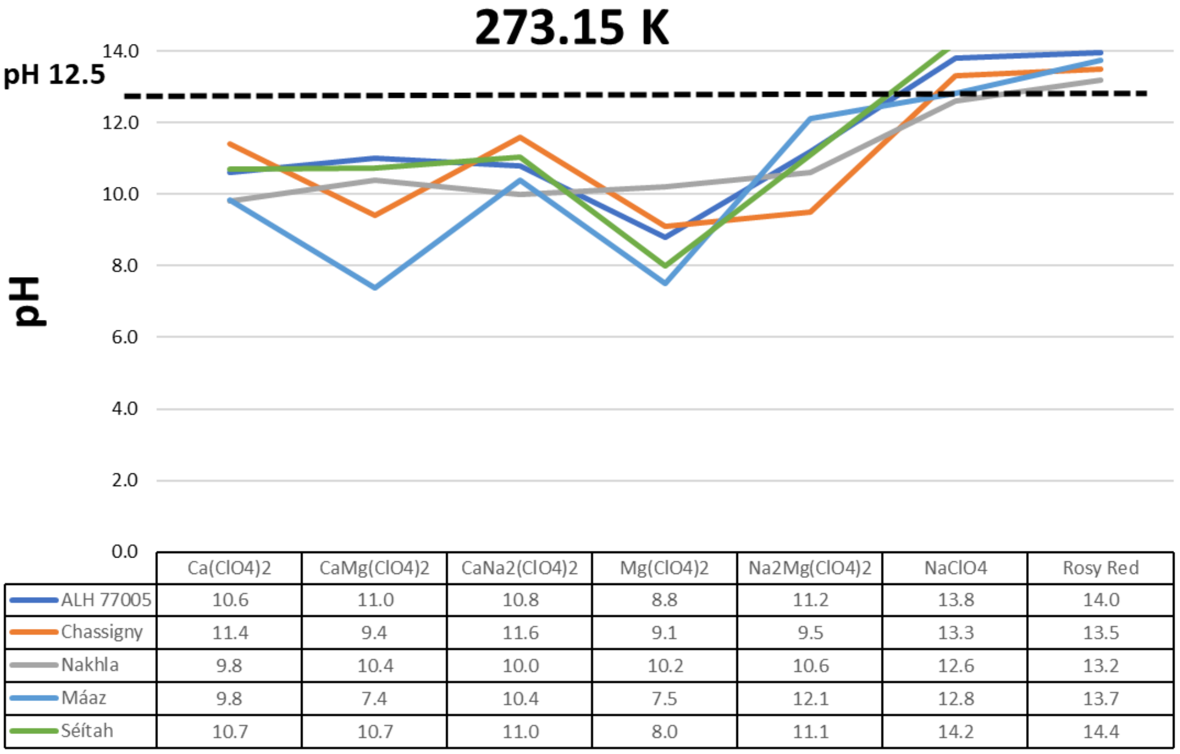

	Ca(ClO4)2	CaMg(ClO4)2	CaNa2(ClO4)2	Mg(ClO4)2	Na2Mg(ClO4)2	NaClO4	Rosy Red
ALH 77005	10.6	11.0	10.8	8.8	11.2	13.8	14.0
Chassigny	11.4	9.4	11.6	9.1	9.5	13.3	13.5
Nakhla	9.8	10.4	10.0	10.2	10.6	12.6	13.2
Máaz	9.8	7.4	10.4	7.5	12.1	12.8	13.7
Séítah	10.7	10.7	11.0	8.0	11.1	14.2	14.4

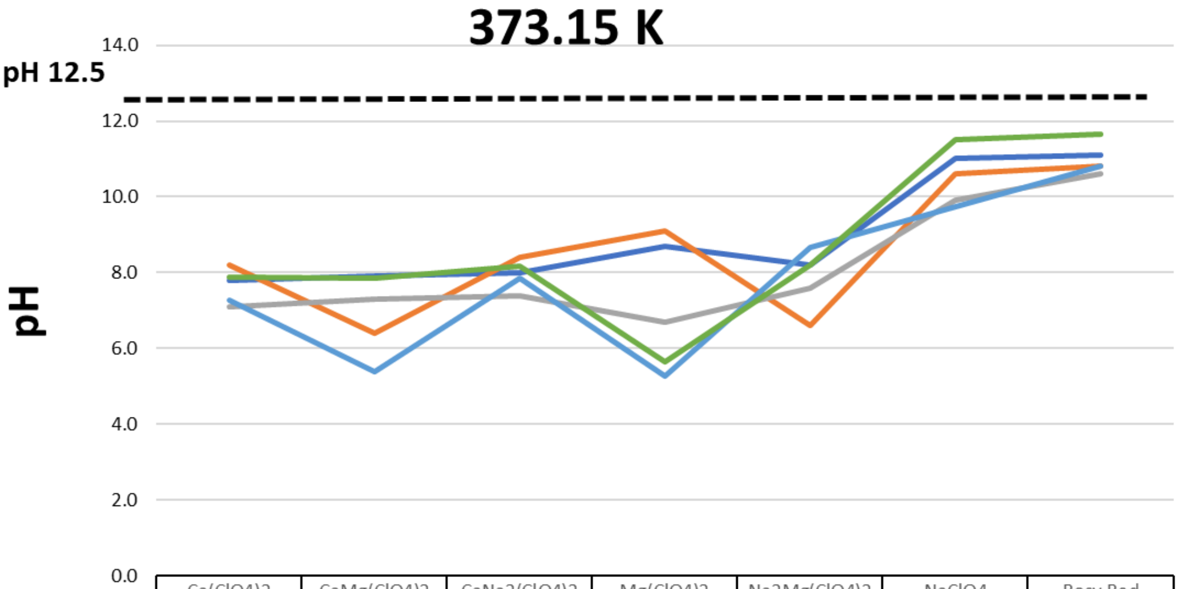

	Ca(ClO4)2	CaMg(ClO4)2	CaNa2(ClO4)2	Mg(ClO4)2	Na2Mg(ClO4)2	NaClO4	Rosy Red
ALH 77005	7.8	7.9	8.0	8.7	8.2	11.0	11.1
Chassigny	8.2	6.4	8.4	9.1	6.6	10.6	10.8
Nakhla	7.1	7.3	7.4	6.7	7.6	9.9	10.6
Máaz	7.3	5.4	7.9	5.3	8.7	9.7	10.8
Séítah	7.9	7.8	8.2	5.7	8.2	11.5	11.7

Supplemental Figure 1. Model simulation pH values. Terrestrial serpentinizing systems show pH values up to approximately 13. Please note that cellular stress in ultrabasic waters is problematic at or around pH 12.5 indicated by the horizontal dashed line.
