# Supplementary material for: Mineral Indicators of Geologically Recent Past Habitability on Mars
Source: Life (Basel). 2023 Dec 15;13(12):2349. doi: 10.3390/life13122349 (PMC10744562; doi:10.3390/life13122349)
Supplement: Supplementary file 1 [file life-13-02349-s001.zip › Supplementary Table_2.pdf]

	ALH 77005	Chassigny	Nakhla	Máaz	Séítah
Major oxide (w.t. %)					
**MgO**	28.2	31.8	12.1	2.28	19.24
**Al2O3**	2.87	0.72	1.68	8.8	2.25
**SiO2**	42.4	37.4	48.6	48.81	40.42
**CaO**	3.16	0.66	14.7	7.85	3.15
**TiO2**	0.39	0.08	0.34	2.44	0.39
**Na2O(ox)**	0.47	0.12	0.46	3.97	1.32
**K2O(ox)**	0.03	0.036	0.13	1.11	0.15
**Cr2O3**	0.98	0.77	0.26	0.01	0.3
**MnO**	0.45	0.53	0.49	0.48	0.7
**FeO(ox)**	20.1	27.3	20.6	21.69	30.61
**2O5**	0.4	0.071	0.13	2.57	0.42
**S**	0.051	0.026	0.026	0	0
**H2O**	0	0.07	0.057	0	0
**Geochemical classification**	Ultramafic	Ultramafic	Mafic	Mafic	Ultramafic
Prominent Mineral constituents	*Olivine, pyroxene, Maskelynite, opaques*	*Olivine, pyroxene, plagioclase, chromite, ilmenite*	*Pyroxene, Olivine, Mesostasis*	*Augite, plagioclase, ilmenite, ferrosilite, and unidentified iron oxide solids*	*Olivine, augite, with minor amounts of feldspars, phosphates, Cr- and Ti-bearing Fe oxide solids*
References	*Lodders, 1998*	*Lodders, 1998*	*Lodders, 1998*	*Farley et al., 2022*	*Farley et al., 2022*

Supplementary Table 2. Meteorite and data for Máaz and Séítah sites were input as average whole-rock compositions, ALH77005 (lherzolitic shergottite, Lodders (1998)), Chassigny (type chassignite, Lodders (1998)), Nakhla (type nakhlite, Lodders (1998)), Perseverance sites, Máaz and Séítah (Farley et al. 2022).
