# Supplementary figures and images for: Mineral Indicators of Geologically Recent Past Habitability on Mars
Source: Life (Basel). 2023 Dec 15;13(12):2349. doi: 10.3390/life13122349 (PMC10744562; doi:10.3390/life13122349)

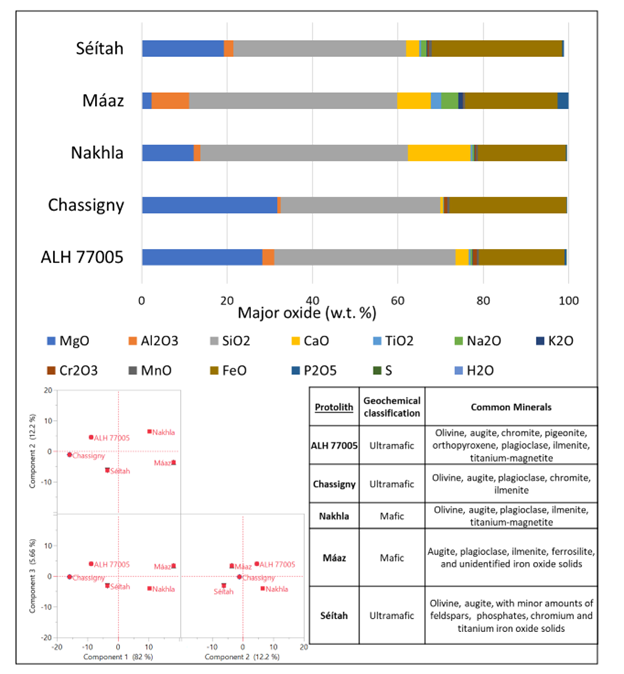

Supplement: Supplementary file 1 [file life-13-02349-s001.zip › Supplemental Figure 2.png]
